# Role of innate lymphoid cells in chronic colitis during anti-IL-17A therapy

**DOI:** 10.1038/s41598-019-57233-w

**Published:** 2020-01-15

**Authors:** Chan Hyuk Park, A-reum Lee, Sang Bong Ahn, Chang Soo Eun, Dong Soo Han

**Affiliations:** 1 Department of Internal Medicine, Hanyang University Guri Hospital, Hanyang University College of Medicine, Guri, Korea; 2 Department of Internal Medicine, Hanyang University Guri Hospital, Hanyang University College of Medicine, Guri, Korea; 3 Department of Internal Medicine, Nowon Eulji Medical Center, Eulji University, Seoul, Korea; 4 Department of Internal Medicine, Hanyang University Guri Hospital, Hanyang University College of Medicine, Guri, Korea; 5 Department of Internal Medicine, Hanyang University Guri Hospital, Hanyang University College of Medicine, Guri, Korea

**Keywords:** Innate immunity, Crohn's disease

## Abstract

IL-17A is an important cytokine in intestinal inflammation. However, anti-IL-17A therapy does not improve clinical outcomes in patients with Crohn’s disease. We aimed to evaluate the role of RORγt^+^ innate lymphoid cells (ILCs) in murine colitis models in the absence of IL-17A. An acute colitis model was induced with either dextran sulfate sodium (DSS) or trinitrobenzenesulfonic acid (TNBS) and a chronic colitis model was induced by CD4^+^CD45RB^hi^ T cell transfer from either wild-type C57BL/6 or *Il17a*^−/−^ mice. An anti-IL-17A antibody, secukinumab, was also used to inhibit IL-17A function in the colitis model. Flow cytometry was performed to analyze the population of RORγt^+^ ILCs in the colonic lamina propria of mice with chronic colitis. Acute intestinal inflammation due to DSS and TNBS was attenuated in IL-17A knockout mice, whereas chronic colitis was not relieved by T cell transfer from *Il17a*^−/−^ mice (% of original body weight: wild-type mice vs. *Il17a*^−/−^ mice, 81.9% vs. 82.2%; *P* = 0.922). However, the mean proportion of Lin^-^RORγt^+^ lymphocytes was higher after T cell transfer from *Il17a*^−/−^ mice than that after T cell transfer from wild-type mice (28.8% vs. 18.5%). The proportion of Lin^-^RORγt^+^ was also increased in *Rag2*^−/−^ mice that received T cell transfer from wild-type mice when anti-IL-17A antibody was administered (31.7%). Additionally, *Il6* and *Il22* tended to be highly expressed after T cell transfer from *Il17a*^−/−^ mice. In conclusion, RORγt^+^ ILCs may have an important role in the pathogenesis of chronic colitis in the absence of IL-17A. Blocking the function of IL-17A may upregulate *Il6* and recruit RORγt^+^ ILCs in chronic colitis, thereby upregulating IL-22 and worsening the clinical outcomes of patients with Crohn’s disease.

## Introduction

In patients with Crohn’s disease, IL-17A-producing cells are highly prevalent in the intestinal mucosa, and intestinal mucosal cells exhibit high transcript expression levels of IL-17A^1,2^. Fecal IL-17A has also been observed in patients with active Crohn’s disease. Based on these findings, anti-IL-17A therapy would be expected to be effective in treating patients with Crohn’s disease^3^. However, initial trials of anti-IL-17A therapy for Crohn’s disease has yielded disappointing results. In a clinical trial of anti-IL-17A antibody, secukinumab, for Crohn’s disease did not improve symptoms^3^. Moreover, severe adverse events, including cases of Crohn’s disease worsening further, occurred in the secukinumab group. A phase II trial of brodalumab, an IL-17R blocker, also showed no benefit for Crohn’s disease compared to placebo^4^. In the context of the successful outcomes of secukinumab treatment for psoriasis and rheumatoid arthritis^5–7^, the lack of efficacy for secukinumab in treating Crohn’s disease was highly disappointing. Unlike the negative results of anti-IL-17A therapy, treatment with IL-12/IL-23 antagonists or selective IL-23 antagonists, including ustekinumab, risankizumab, and brazikumab, which block the upstream pathway of IL-17, are effective against Crohn’s disease^8–10^.

In the phase 3 trials of secukinumab in plaque psoriasis and psoriatic arthritis, two patients developed Crohn’s disease after treatment with secukinumab. Moreover, a case of rapid onset of fulminant inflammatory bowel disease after a single secukinumab infusion has been reported^11,12^. We hypothesized that innate immunity influences the response to anti-IL-17A therapy for Crohn’s disease, since RORγt^+^ ILCs are primarily distributed in the intestine rather than the skin or joints^13,14^.

Several hypotheses have been proposed to explain the lack of efficacy of anti-IL-17A therapy in treating Crohn’s disease; these hypotheses have included the complexity of Th17 biology, the role of IL-17A in yeast immunity, and the action of IL-17A on intestinal epithelium to promote barrier function^15–17^. However, Th17 cells’ complex polarity and epithelial barrier integrity may not be the only possible explanation for the failure of anti-IL-17A therapy in Crohn’s disease, because RORγt^+^ innate lymphoid cells (ILCs) also produce IL-17A^18^. Moreover, RORγt^+^ ILCs are almost exclusively found in the intestinal lamina propria and have been suggested to play a crucial role in the pathogenesis of Crohn’s disease^13^. If anti-IL-17A therapies influence the development of RORγt^+^ ILCs, these therapies might have unintended outcomes in patients with chronic colitis that are distinct from those in patients with psoriasis.

Therefore, we aimed to evaluate the impact of blocking IL-17A function in acute and chronic colitis mouse models; to this end, we utilized *Il17a*^−/−^ knockout mice. Subsequently, acute and chronic colitis mouse models were validated using the IL-17A inhibitor secukinumab. We also assessed whether RORγt^+^ ILCs are recruited in the absence of IL-17A in our chronic colitis mouse model.

## Results

### Role of IL-17A in the acute colitis model

**Figure 1:**
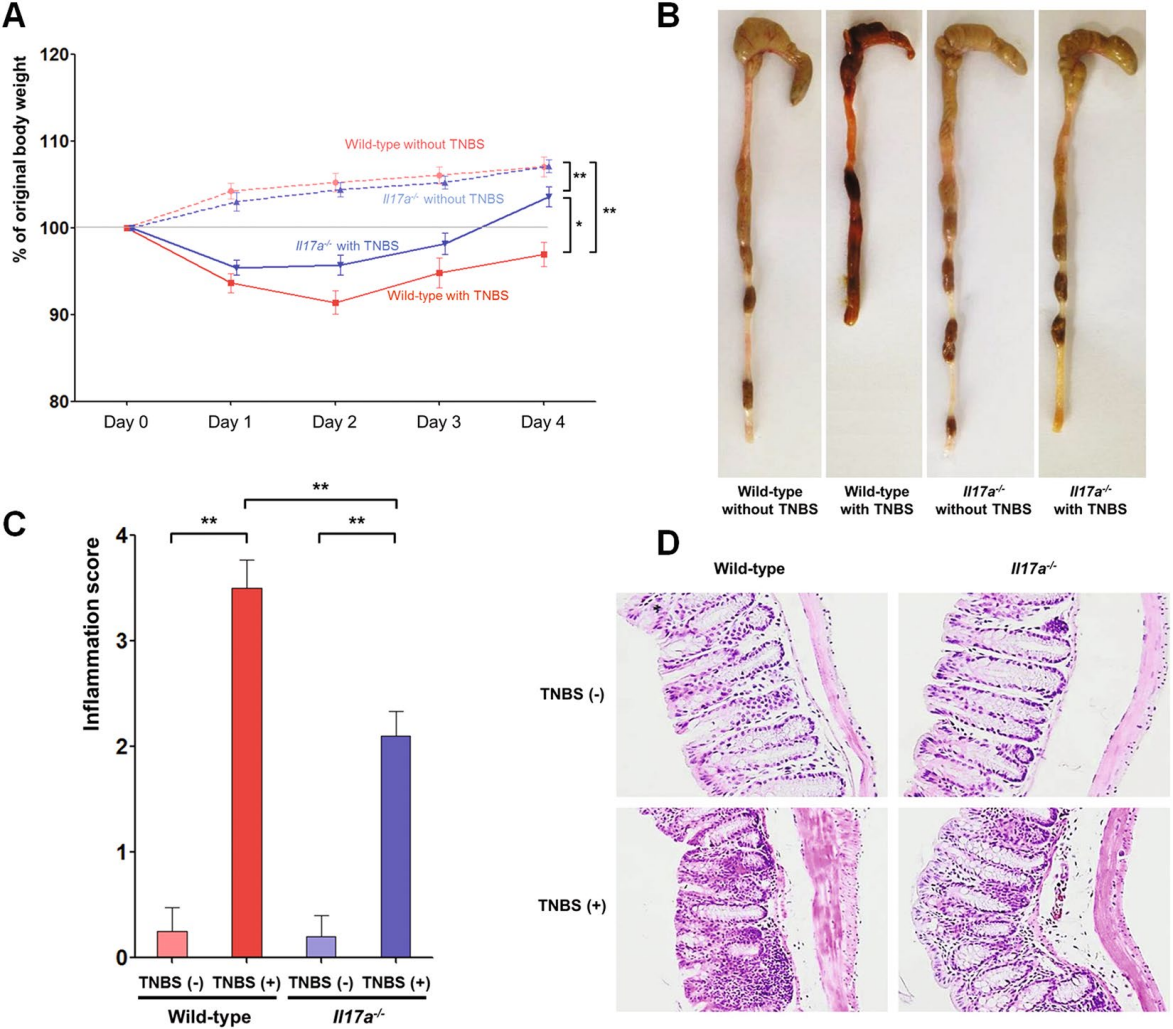
TNBS-induced colitis model. Bodyweight changes (**A**), representative gross photos of the colorectal area (**B**), inflammation scores (**C**), and representative microscopic images of histopathological examination (x200) (**D**) of wild-type and *Il17a*^−/−^ mice (wild-type mice without TNBS: n = 4; wild-type mice with TNBS: n = 8; *Il17a*^−/−^ mice without TNBS: n = 5; *Il17a*^−/−^ mice with TNBS: n = 10). Bars represent standard errors. * *P* < 0.05, ** *P* < 0.01. TNBS, 2,4,6-trinitrobenzenesulfonic acid.

To investigate the role of IL-17A in acute intestinal inflammation, 3.7 mg of trinitrobenzenesulfonic acid (TNBS) was administered to wild-type (WT) and *Il17a*^−/−^ mice (Fig. 1). After TNBS treatment, body weight decreased on the first and second days in both groups. The mean body weight of WT and *Il17a*^−/−^ mice that received TNBS were lower than those that did not (WT, *P* < 0.001; *Il17a*^−/−^, *P* < 0.001; Fig. 1A). However, among the mice that received TNBS, *Il17a*^−/−^ mice tended to be heavier than WT mice (*P* = 0.020). In addition, the mean total colon length of *Il17a*^−/−^ mice was longer than that of WT mice (Fig. 1B). Although macroscopic inflammation scores increased after TNBS administration in both WT and *Il17a*^−/−^ mice (WT, *P* < 0.001; *Il17a*^−/−^, *P* < 0.001), the post-TNBS intestinal inflammation scores of the *Il17a*^−/−^ mice were lower than those of the WT mice (*P* = 0.001; Fig. 1C).

**Figure 2:**
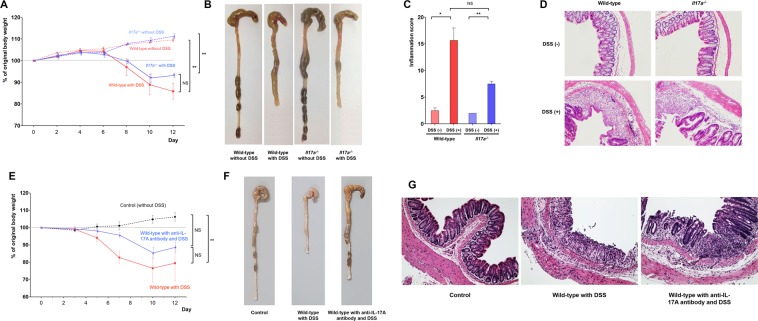
DSS-induced colitis model. Bodyweight changes (**A**), representative gross photos of the colorectal area (**B**), inflammation scores (**C**), and representative microscopic images of histopathological examination (x200) (**D**) after administration of DSS for wild-type and *Il17a*^−/−^ mice (wild-type mice without DSS: n = 5; wild-type mice with DSS: n = 8; *Il17a*^−/−^ mice without DSS: n = 7; *Il17a*^−/−^ mice with DSS: n = 9). Bodyweight changes (**E**), representative gross photos of the colorectal area (**F**), and representative microscopic images of histopathological examination (x200) (**G**) according to administration of anti-IL-17A antibody (wild-type mice without DSS: n = 3; wild-type mice with DSS: n = 3; wild-type mice with anti-IL-17A antibody and DSS: n = 4). In the anti-IL-17A antibody group, 100 μL of secukinumab was injected intraperitoneally three times per week starting two weeks before DSS administration, continuing until the end of the experiment. Bars represent standard errors. * *P* < 0.05, ** *P* < 0.01. DSS, dextran sulfate sodium; NS, not significant.

Next, we compared colonic inflammation between WT and *Il17a*^−/−^ mice in the dextran sodium sulfate (DSS)-induced colitis model (Fig. 2). On the 8^th^ day after DSS treatment, the body weight of both WT and *Il17a*^−/−^ mice began to decrease. However, *Il17a*^−/−^ mice tended to lose less weight than WT mice (*P* = 0.442; Fig. 2A). The total colon length of WT and *Il17a*^−/−^ mice was similar (Fig. 2B). Microscopic examination of H&E-stained colonic tissue specimens revealed that the intestinal inflammation scores of the *Il17a*^−/−^ mice after DSS administration tended to be lower than those in the WT mice after DSS administration (*P* = 0.074; Fig. 2C). The validation study using anti-IL-17A antibody showed similar results to the DSS-induced colitis model in *Il17a*^−/−^ mice (Fig. 2E,F). WT mice that received anti-IL-17A antibody tended to lose less weight than those who did not (Fig. 2E).

### Role of IL-17A in the chronic colitis model

**Figure 3:**
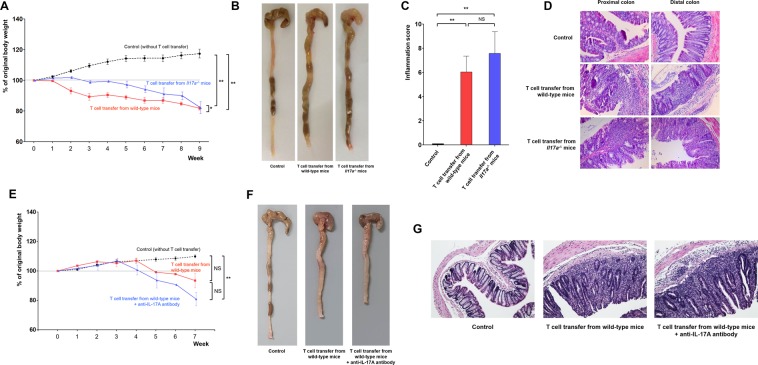
CD4^+^CD45RB^hi^ T cell transfer-induced colitis model. Bodyweight changes (**A**), representative gross photos of the colorectal area (**B**), inflammation scores (C), and representative microscopic images of histopathological examination (x200) (**D**) after T cell transfer from wild-type or *Il17a*^−/−^ mice (*Rag2*^−/−^ mice without T cell transfer: n = 13; *Rag2*^−/−^ mice with T cell transfer from wild-type mice: n = 8; *Rag2*^−/−^ mice with T cell transfer from *Il17a*^−/−^ mice: n = 8). Bodyweight changes (**E**), representative gross photos of the colorectal area (**F**), representative microscopic images of histopathological examination (x200) (**G**) after T cell transfer from wild-type mice according to administration of anti-IL-17A antibody (*Rag2*^−/−^ mice without T cell transfer [control]: n = 3; *Rag2*^−/−^ mice received T cell transfer from wild-type mice: n = 2; *Rag2*^−/−^ mice received T cell transfer from wild-type mice and anti-IL-17A antibody: n = 2). In the anti-IL-17A antibody group, 100 μL of secukinumab was injected intraperitoneally three times per week from the time of T cell transfer to the end of the experiment. Bars represent standard errors. *P < 0.05, **P < 0.01. NS, not significant.

To determine the effects of blocking the function of IL-17A in chronic colitis, we induced colonic inflammation in *Rag2*^−/−^ mice via a transfer of CD4^+^CD45RB^hi^ T cells isolated from WT or *Il17a*^−/−^ mice. As shown in Fig. 3A, the body weight of *Rag2*^−/−^ mice that received T cells from WT mice decreased starting the 2^nd^ week after the transfer. In contrast, *Rag2*^−/−^ mice that received T cells from *Il17a*^−/−^ mice maintained their weight until the 4^th^ week, although they also presented with lower body weight than control mice (*Rag2*^−/−^ mice that did not receive T cells). Throughout the monitoring period (week 0 to week 9), the mean body weight of mice that received T cells from *Il17a*^−/−^ mice was higher than that of mice that received T cells from WT mice (*P* = 0.012 by repeated measures analysis of variance (ANOVA)). Eventually, however, the body weight of *Rag2*^−/−^ mice that received T cells from *Il17a*^−/−^ mice decreased from the 6^th^ week onward. Nine weeks after T cell transfer, body weight did not differ between mice that received T cells from WT mice and those that received T cells from *Il17a*^−/−^ mice (% of original body weight: 81.9% vs. 82.2%; *P* = 0.922). The total colon length was also similar between mice that received T cells from WT mice and mice that received T cells from *Il17a*^−/−^ mice (Fig. 3B). In addition, the intestinal inflammation scores of H&E-stained colonic tissue specimens from the two groups were not significantly different (*P* = 0.494; Fig. 3C).

In the validation study using anti-IL-17A antibody, chronic colitis was induced in all the *Rag2*^−/−^ mice that received T cells (Fig. 3E–G). Although statistical significance was not achieved in the comparison between the T cell transfer with anti-IL-17A antibody group and the group that did not receive anti-IL-17A antibody due to a small sample size, administration of anti-IL-17A antibody could not attenuate chronic colitis.

These data indicate that blocking IL-17A function did not prevent chronic colitis, although we noted that induction of colitis was delayed in mice that received T cells from *Il17a*^−/−^ mice. To compare the difference between T cell transfer from WT mice and that from *Il17a*^−/−^ mice, we analyzed the expression levels of various inflammatory cytokines including IL-12, IFN-γ, and IL-6 in colonic tissue samples from *Rag2*^−/−^ mice via enzyme-linked immunosorbent assay (ELISA) (Fig. S1). Although none of the differences were statistically significant, all cytokines tended to show higher expression after T cell transfer (from both WT and *Il17a*^−/−^ mice). The expression levels of IL-12 and IFN-γ, which are associated with Th1 and group 1 ILC differentiation, were similar between mice that received T cells from WT versus *Il17a*^−/−^ mice (IL-12p40: 837.8 ± 183.3 pg/mL vs. 830.9 ± 216.4 pg/mL, *P* = 0.982; IFN-γ: 205.4 ± 34.4 pg/mL vs. 147.2 ± 57.7 pg/mL, *P* = 0.416). However, expression of IL-6, which is associated with Th17 and RORγt^+^ ILC differentiation, tended to be higher in mice that received T cells from *Il17a*^−/−^ mice than in mice that received T cells from WT mice (T cell transfer from WT mice vs. T cell transfer from *Il17a*^−/−^ mice: 14.7 ± 4.0 pg/mL vs. 28.9 ± 6.1 pg/mL, *P* = 0.134).

### Innate lymphoid cells in the chronic colitis model

**Figure 4:**
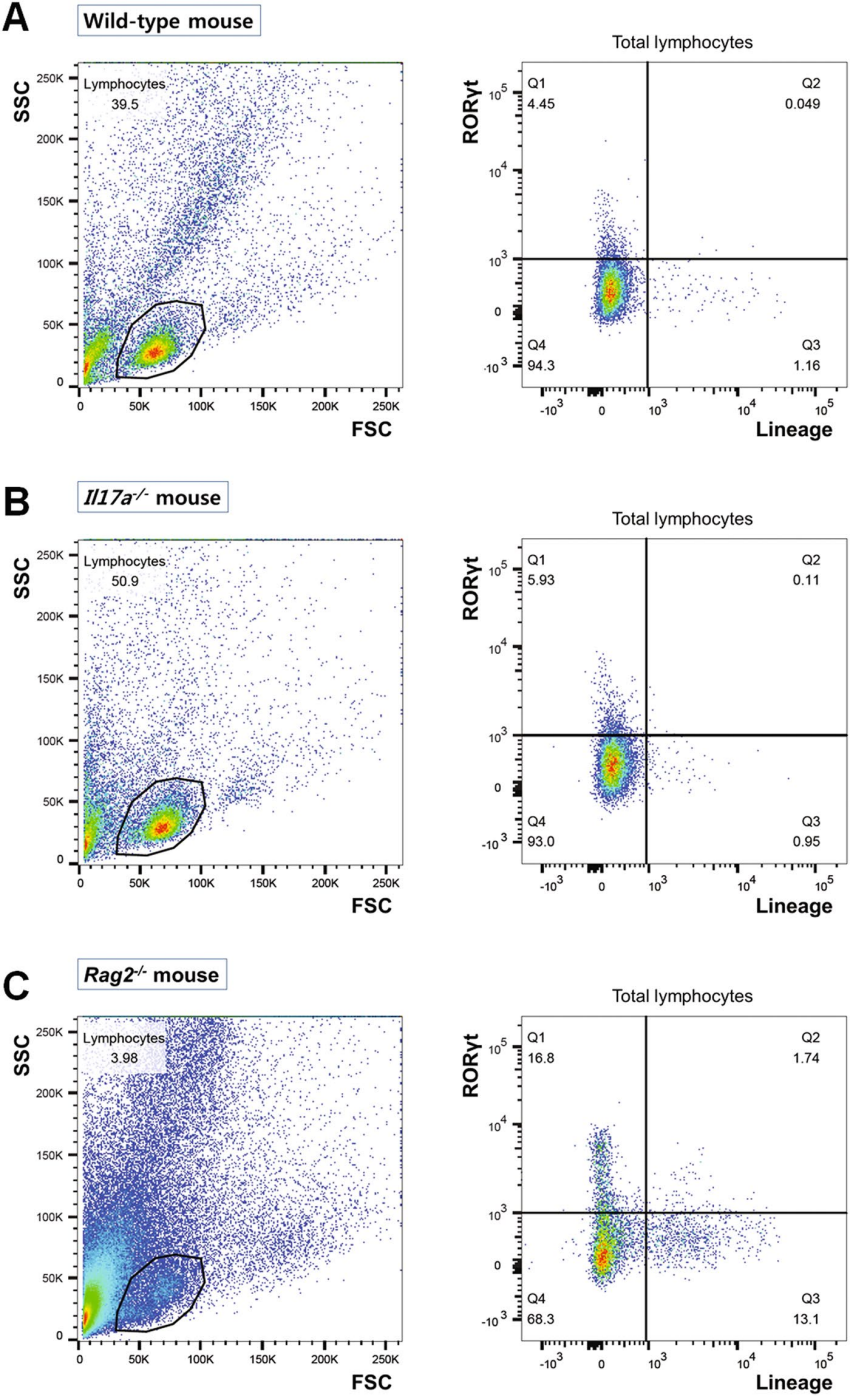
Flow cytometry analysis of colonic lamina propria lymphocytes in wild-type C57BL/6 mice (**A**), *Il17a*^−/−^ mice (**B**), and *Rag2*^−/−^ mice (**C**). FSC, forward scatter; SSC, side scatter.

To evaluate ILC changes in the chronic colitis model, we analyzed colonic lamina propria cells from WT, *Il17a*^−/−^, and *Rag2*^−/−^ mice with flow cytometry. As shown in Fig. 4, relatively small populations of RORγt^+^ cells were observed in WT and *Il17a*^−/−^ mice (4.5% and 6.0%, respectively). Of particular note, the percentages of Lin^-^RORγt^+^ cells [which include group 3 ILCs (LTi cells and ILC3s)] were 4.5% and 5.9% in WT and *Il17a*^−/−^ mice, respectively. In the absence of adaptive immunity (i.e. in *Rag2*^−/−^ mice), the proportion of RORγt^+^ ILCs was higher than in WT or *Il17a*^−/−^ mice (WT vs. *Il17a*^−/−^ vs. *Rag2*^−/−^ mice, 4.5% vs. 5.9% vs. 16.8%; Fig. 4C), which is consistent with a previous study^19^.

**Figure 5:**
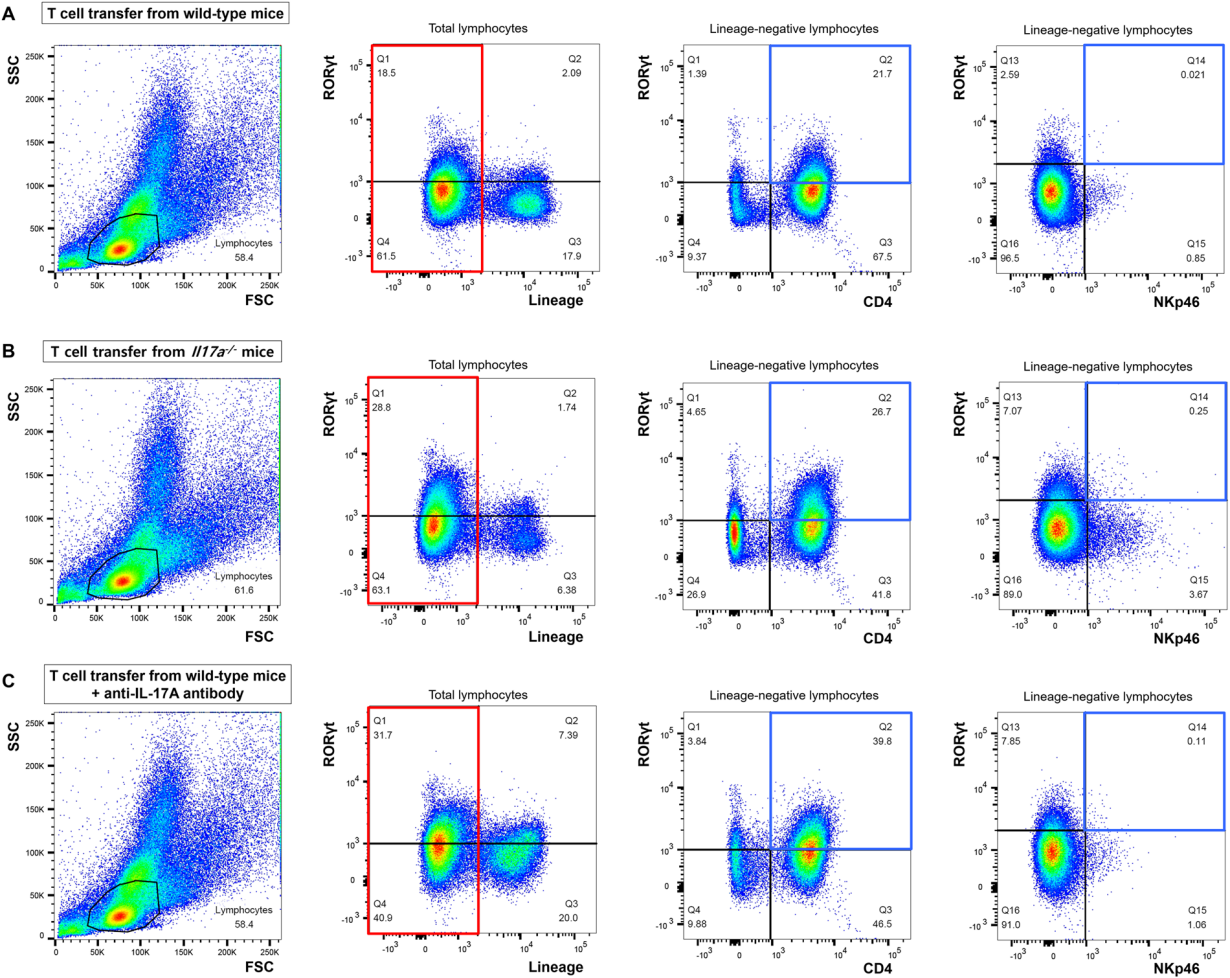
Flow cytometry analysis of colonic lamina propria lymphocytes in *Rag2*^−/−^ mice that received T cell transfers from wild-type mice (**A**) or *Il17a*^−/−^ mice. (**B**) Flow cytometry analysis was performed on the 9^th^ week after the T cell transfer. FSC, forward scatter; SSC, side scatter.

After transferring CD4^+^CD45RB^hi^ T cells from WT mice, the proportion of Lin^-^RORγt^+^ lymphocytes was similar to that in *Rag2*^−/−^ mice that did not receive T cells (T cell transfer from WT mice vs. without T cell transfer: 18.5% vs. 16.8%, Figs. 4C and 5A). The majority of RORγt^+^ ILCs expressed CD4 and did not express NKp46 (Fig. 5A). These results show that, in the absence of adaptive immunity, most RORγt^+^ ILCs were CD4^+^LTi cells, while CD4^-^LTi cells and ILC3s represented only a minor portion of ILCs.

In mice that received T cells from *Il17a*^−/−^ mice, the proportion of Lin^-^RORγt^+^ lymphocytes increased to 28.8% (Fig. 5B). The majority of RORγt^+^ ILCs also expressed CD4 and did not express NKp46 (T cell transfer from WT mice vs. T cell transfer from *Il17a*^−/−^ mice: CD4^+^RORγt^+^ ILCs, 21.7% vs. 26.7%; NKp46^+^RORγt^+^ ILCs, 0.02% vs. 0.25%). However, the proportion of CD4^-^RORγt^+^ ILCs was also slightly elevated compared to the proportion of mice that received T cells from WT mice (T cell transfer from WT mice vs. T cell transfer from *Il17a*^−/−^ mice: 1.4% vs. 4.7%). In addition, the proportion of CD4^-^RORγt^-^ ILCs, which included ILC1s, was increased in *Rag2*^−/−^ mice that received T cells from *Il17a*^−/−^ mice compared to mice that received T cells from WT mice (T cell transfer from WT mice vs. T cell transfer from *Il17a*^−/−^ mice: CD4^-^RORγt^-^ ILCs, 9.4% vs. 26.9%). Overall, RORγt^+^ ILCs and ILC1s were more prevalent in *Rag2*^−/−^ mice that received T cells from *Il17a*^−/−^ mice than in mice that received T cells from WT mice.

Findings were similar in experiment using anti-IL-17A antibody (Fig. 5C). RORγt^+^ ILCs were observed in large numbers in *Rag2*^−/−^ mice that received T cells from WT mice when anti-IL-17A antibody was administered. Furthermore, the majority of RORγt^+^ ILCs expressed CD4 (39.8%), but they barely expressed NKp46 (0.1%).

### Innate lymphoid cells in the spleens of mice with chronic colitis

To determine whether ILCs were exclusively enriched in the intestine, we used flow cytometry to analyze spleen cells from mice with T cell transfer-induced colitis. As shown in Fig. S2, the lymphocyte proportions of RORγt^+^ ILCs were similar among *Rag2*^−/−^ mice that did not receive T cells, *Rag2*^−/−^ mice that received T cells from WT mice, and *Rag2*^−/−^ mice that received T cells from *Il17a*^−/−^ mice (6.4% vs. 7.6% vs. 5.2%, respectively). Additionally, the lineage-negative lymphocyte proportions of CD4^+^RORγt^+^ ILCs were similar between *Rag2*^−/−^ mice that received T cells from WT mice and those that received T cells from *Il17a*^−/−^ mice (4.6% vs. 4.5%).

### Innate lymphoid cells in the colonic lamina propria with acute colitis model

To determine whether the increase in the proportion of Lin^-^RORγt^+^ lymphocytes is a characteristic finding in the chronic colitis model, we analyzed colonic lamina propria cells from WT and *Il17a*^−/−^ mice with and without DSS treatment. As shown in Fig. S3, only small populations of RORγt^+^ cells were observed in the acute colitis model (both WT and *Il17a*^−/−^ mice [1.1% and 2.0%, respectively]).

### Transcript expression in mice with chronic colitis

**Figure 6:**
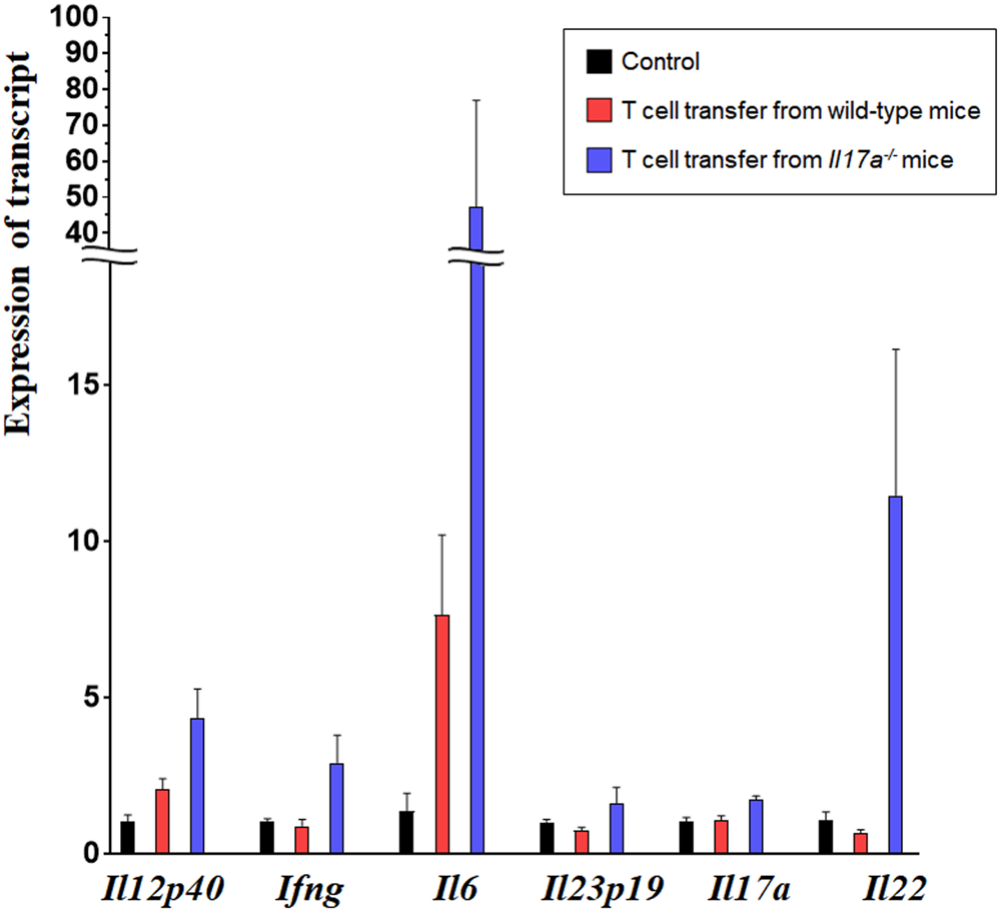
Expression of transcripts in colon tissue from mice with T cell-induced colitis. The relative expression of each transcript was measured by quantitative real-time polymerase chain reaction and calculated with the 2^−*Δ ΔCT*^ method using β-actin levels for normalization (*Rag2*^−/−^ mice without T cell transfer: n = 3; *Rag2*^−/−^ mice with T cell transfer from wild-type mice: n = 3; *Rag2*^−/−^ mice with T cell transfer from *Il17a*^−/−^ mice: n = 3). There was no significant difference between any of the groups. Although statistical power was insufficient, *Il6* and *Il22* tended to be higher in *Rag2*^−/−^ mice with T cell transfer from *Il17a*^−/−^ mice than those with T cell transfer from wild-type mice. Bars represent standard errors. All reactions were performed in duplicate.

The transcript expression levels of various cytokines, including IL-12, IFN-γ, IL-6, Il-23, IL-17, and IL-22 were evaluated next via quantitative real-time polymerase chain reaction (Fig. 6). Although statistical significance was not reached due to a limited sample size, *Il6* and *Il22*, which are involved in Th17 and RORγt^+^ ILC differentiation, tended to be highly expressed in mice that received T cells from *Il17a*^−/−^ mice. Additionally, *Il12p40 and Ifng* also showed a trend of high expression in mice that received T cells from *Il17a*^−/−^ mice, even though IL-12 and IFN-γ are more closely related to Th1 and ILC1 differentiation than Th17 and RORγt^+^ ILC differentiation. Since a subpopulation of RORγt^+^ ILCs may downregulate RORγt and acquire the capacity to produce IFNγ^20^, these findings imply that innate immunity (especially RORγt^+^ ILCs) is involved in the pathogenesis of T cell transfer-induced colitis.

## Discussion

We found that the absence of IL-17A tended to attenuate acute colitis. These results are consistent with previous studies of TNBS-induced and DSS-induced colitis models, both of which demonstrated that IL-17A plays a pathogenic role in acute colitis^17,21^. In contrast, the severity of chronic colitis was not affected by T cell transfer from *Il17a*^−/−^ mice or anti-IL-17A therapy^22,23^. Our study also demonstrated that blocking IL-17A function did not attenuate chronic colitis. In addition, we found that weight loss in mice that received T cells from *Il17a*^−/−^ mice occurred later than in mice that received T cells from WT mice.

One of the possible reasons for the difference in the outcome of anti-IL-17A therapy between acute and chronic colitis models may be the different effects of cytokines depending on the phase of colitis. It has been shown that the mucosal pattern of effector cytokines differs according to the different stages of Crohn’s disease^24^. For example, in the early stages of Crohn’s disease, macroscopically unaffected neoterminal ileum contained high levels of IFN-γ and IL-21, which are produced by Th1 cells. On the contrary, in the advanced lesions of Crohn’s disease, marked up-regulation of IL-17A and induction of IL-23 and IL-6 were observed.

In the current study, we hypothesize that the delay in colitis development between mice that received T cells from WT mice and those that received T cells from *Il17a*^−/−^ mice was due to the differentiation of ILCs that are involved in chronic colitis in the absence of IL-17A. Anti-IL-17A therapy can attenuate acute colitis because IL-17A is a potent inducer of neutrophil-promoting cytokines. In cases of chronic colitis, however, disruption of epithelial barrier integrity and activation of ILC may worsen disease activity due to treatment with anti-IL-17A therapy. Although weight loss was similar at the 9^th^ week between the two chronic colitis mouse models (T cell transfer from WT mice vs. T cell transfer from *Il17a*^−/−^ mice), the proportions of ILCs in the intestinal lamina propria were different. In particular, the proportion of RORγt^+^ ILCs was increased in the absence of IL-17A. Additionally, the majority of RORγt^+^ ILCs were CD4^+^LTi cells. In the intestinal lamina propria, CD4^+^LTi cells may play a major role in the development of chronic colitis in the absence of IL-17A. In addition, *Il6* and *Il22*, which are involved in LTi cell differentiation^13^, were highly expressed in mice that received T cells from *Il17a*^−/−^ mice. The elevated expression of *Il6* in the absence of IL-17A is particularly noteworthy because IL-6 is a critical cytokine that promotes IL-17A production in both humans and mice^13,25^. RORγt^+^ ILCs (which include LTi cells) respond to IL-6 and produce the effector cytokine IL-22. Thus, a lack of IL-17A may upregulate IL-6 and recruit RORγt^+^ ILCs via negative feedback mechanisms; thus, IL-22 can be upregulated and worsen the clinical outcomes of patients with Crohn’s disease^13^. In order to prevent this unintended response of ILC, simultaneous blocking of IL-17A and IL-6 may be considered as a potential strategy in the treatment of Crohn’s disease.

Although IFN-γ is an effector cytokine of ILC1s and not RORγt^+^ ILCs, we observed a trend of high expression of *Ifng* in the mice that received T cells from *Il17a*^−/−^ mice. An increase of IFN-γ may be caused by the activation of pathogenic T cells; however, we proposed that IFN-γ would be increased by ILC1s in the absence of IL-17A. In our study, we found that the proportions of ILCs other than RORγt^+^ ILCs (e.g., ILC1s) were also increased in mice with chronic colitis in the absence of IL-17A. Although the majority of the elevated ILCs were CD4^+^LTi cells that belonged to the RORγt^+^ ILC group, a subpopulation of RORγt^+^ ILCs may downregulate RORγt and gain the ability to produce IFNγ^20^. Our flow cytometry analysis showed that Lin^-^RORγt^-^ cells, which included ILC1s, were more abundant in *Rag2*^−/−^ mice that received T cells from *Il17a*^−/−^ mice than in those that received T cells from WT mice. Immune cell plasticity is a distinctive characteristic in adaptive immunity as well as innate immunity. Previous studies have demonstrated that Tregs can be transdifferentiated from Th17 cells by myeloid-derived suppressor cells^26–28^. Our results indicate that blocking IL-17A function may also increase the population of RORγt^+^ ILCs (which include LTi cells) via IL-6 production. Moreover, some RORγt^+^ ILCs may transform into ILC1s. RORγt^+^ ILCs and transformed ILC1s may eventually worsen intestinal inflammation via IL-22 and IFN-γ, respectively, in chronic colitis when IL-17A is absent. Therefore, additional targets besides IL-17A are required to control intestinal inflammation in chronic colitis. As mentioned above, we propose that IL-6 as a potential target for Crohn’s disease. A study reporting a pilot randomized trial of anti-IL-6R monoclonal antibodies suggested a clinical effect in active Crohn’s disease^29^, which supports our theory.

In addition, we demonstrated that the proportion of RORγt^+^ ILCs in spleen lymphocytes did not increase in mice with T cell transfer-induced colitis. Since ILCs are distributed in a tissue-specific manner, the effects of ILC differentiation may drive different outcomes according to the type of disease. These characteristics of ILCs may explain the different outcomes of anti-IL-17A therapy in the context of psoriasis, RA, and Crohn’s disease, each of which involves different organs.

Additionally, we identified that an increase in the proportion of RORγt^+^ ILCs was observed only in the chronic colitis model and not in the acute colitis model. Anti-IL-17A therapy in patients with chronic colitis seems to be unable to improve inflammation, perhaps because it may induce RORγt^+^ ILCs.

In addition to the potential causes mentioned above, changes in the gut microbiome may influence the deterioration of chronic colitis after anti-IL-17A therapy. In the previous study, a higher frequency of fungal infection was reported in patients with Crohn’s disease after treatment with secukinumab^3^. If anti-IL-17A therapy affects not only fungi but also the gut microbiome, this may increase disease activity of Crohn’s disease.

In this article, we outlined a potential mechanism by which the unexpected results of anti-IL-17A therapy in Crohn’s disease can be explained, and we suggested potential treatment targets. However, our study did have several limitations. First, we mainly evaluated the outcomes of anti-IL-17A therapy in *Rag2*^−/−^ mice that received T cells from *Il17a*^−/−^ mice. Although we validated the findings using additional experiments with an anti-IL-17A antibody, the sample size of the validation study was limited. However, overall findings were similar between the study with T cell transfer from *Il17a*^−/−^ mice and that with anti-IL-17A antibody. Second, the results for the mRNA profile and flow cytometry were rather descriptive. Although we showed that blocking IL-17A function may be associated with upregulation of *Il6* and recruits RORγt^+^ ILCs in chronic colitis models, the pathogenic relevance of these findings should be investigated further. Moreover, longitudinal measurements of the proportion of ILCs based on the clinical course of the disease after initiating anti-IL-17A therapy may be helpful for understanding ILC differentiation in the context of blocking IL-17A in Crohn’s disease.

Despite these limitations, our study provides a better understanding of the possible mechanisms underlying unresolved intestinal inflammation in the presence of anti-IL-17A therapy in Crohn’s disease. Blocking IL-17A function did not attenuate chronic colitis, although it did reduce intestinal inflammation in acute colitis and in the early phase of chronic colitis. This discrepancy may be caused by Th17 cells heterogeneity or Th17 polarization depending on the stage of intestinal inflammation. Besides, IL-17A blockage may increase the proportion of RORγt^+^ ILCs (which include CD4^+^LTi cells) and ILC1s, thereby eventually worsening chronic colitis. RORγt^+^ ILCs may have an important role in the pathogenesis of chronic colitis in the absence of IL-17A.

## Materials and Methods

### Mice

WT, *Il17a*^−/−^, and *Rag2*^−/−^ C57BL/6 mice were bred and maintained under specific pathogen-free conditions at the accredited animal facilities at Hanyang University. All experiments were performed in accordance with appropriate guidelines for animal experimentation. The experimental protocol was approved by the Institutional Animal Care and Use Committee of Hanyang University.

### Induction of acute colitis

Acute colitis was induced by administering either 3.7 mg of 2,4,6-TNBS in 50% ethanol or 2% DSS. The TNBS- and DSS-induced colitis models were analyzed in separate experiments.

In the TNBS-induced colitis model, animals were fasted overnight for 12 hours prior to hapten TNBS (Sigma-Aldrich, St. Louis, MO) administration. Next, mice were lightly anesthetized using a mixture of 20% v/v isoflurane in propylene glycol. Then, TNBS solution (3.7 mg in 50% ethanol) was administered via an 18 G 1.3 mm diameter IV catheter polyethylene tube. The IV catheter tube was advanced through the rectum into the colon until the tip was 4 cm proximal to the anus. To ensure retention of the haptenating agent within the colon, mice were held in a vertical position for 30 s after TNBS administration to the rectum. Control mice were administered 50% ethanol using the same technique. All mice were sacrificed by cervical dislocation on the 4^th^ day of the experiment.

In the DSS-induced colitis model, DSS (molecular weight 36,000–50,000; MP Biochemicals, Solon, OH) was added to drinking water (2% final concentration) for 5 days. The control group was given the same drinking water without DSS. After 5 days, DSS was removed from the drinking water and the animals received normal drinking water. All mice were sacrificed by cervical dislocation on the 12^th^ day of the experiment.

For the experiments using the acute colitis model, C57BL/6 WT and *Il17a*^−/−^ mice were randomly assigned to either the control or TNBS (or DSS) group. Therefore, each mouse was allocated to one of the following four groups: WT control, WT TNBS (or DSS), *Il17a*^−/−^ control, and *Il17a*^−/−^ TNBS (or DSS).

To validate the results in the *Il17a*^−/−^ mice, we performed additional experiments using the anti-IL-17A antibody secukinumab. In the validation study, WT mice were randomly assigned to one of the following three groups: control, DSS, or anti-IL-17A antibody with DSS. For the anti-IL-17A antibody with DSS group, 100 μL of secukinumab was injected intraperitoneally three times per week starting two weeks before the DSS administration until the end of the experiment.

### Induction of chronic colitis

Chronic colitis was induced via a transfer of naïve CD4^+^CD45RB^hi^ T cells. The naïve CD4^+^CD45RB^hi^ T cells were enriched by nylon passage of spleen cells obtained from C57BL/6 WT or *Il17a*^−/−^ mice and isolated using PE-conjugated anti-CD4 and FITC-conjugated anti-CD45RB monoclonal antibodies (eBioscience, CA) with an FACSAria III Cell Sorting System. The purity of the isolated CD4^+^CD45RB^hi^ T cells was >99%. Purified CD4^+^CD45RB^hi^ T cells (1 × 10^6^ cells) from C57BL/6 WT or *Il17a*^−/−^ mice were injected into *Rag2*^−/−^ mice intraperitoneally. For histopathological analysis of colitis, mice were sacrificed at 9 weeks after the CD4^+^CD45RB^hi^ T cell transfer. The body weight of each mouse was checked every two days during the monitoring period.

The validation study using anti-IL-17A antibody was also performed in the chronic colitis model. WT mice were randomly allocated to one of the following groups: control, T cell transfer, or T cell transfer with anti-IL-17A antibody. Intraperitoneal injection of 100 μL of secukinumab was carried out three times per week starting at the time of T cell transfer until the end of the experiment.

### Assessment of intestinal inflammation

After sacrificing the mice, the colons were removed, and the entire length of each colon was measured. Then, the colon tissue was washed with ice-cold phosphate-buffered saline. The tissue was cut into several segments and then fixed with 10% phosphate-buffered formalin. Tissue segments were embedded in paraffin, stained with hematoxylin and eosin, and assessed under a light microscope.

In the TNBS-induced colitis model, colonic inflammation was assessed based on the following macroscopic scoring criteria for intestinal inflammation^30^: 0 points, no injury; 1 point, focal hyperemia or bowel wall edema with no hemorrhage; 2 points, focal hyperemia or bowel wall edema with hemorrhage at one site; 3 points, extended hyperemia or bowel wall edema with hemorrhage at >1 site; and 4 points, extended hyperemia or bowel wall edema with hemorrhage at >1 site and perforation. In the DSS-induced colitis model, intestinal inflammation was graded based on the following histological grading system of colitis, with a number of points being assigned for each category^31^: (1) inflammation (0–3 points), (2) extent (0–3 points), (3) regeneration (0–4 points), (4) crypt damage (0–4 points), and (5) percent involvement (1–4 points). The total score was the sum of the scores of each category and ranged from 1 to 18. In the CD4^+^CD45RB^hi^ T cell transfer-induced colitis model, intestinal inflammation was scored based on previously reported criteria, as follows^32^: (1) degree of inflammation in the lamina propria (0–3 points), (2) goblet cell loss (0–2 points), (3) abnormal crypts (0–3 points), (4) presence of crypt abscesses (0–1 points), (5) mucosal erosion and ulceration (0–1 points), (6) submucosal spread to transmural involvement (0–3 points), and (7) number of neutrophils counted at ×40 magnification (0–4 points). The total score was calculated by adding the scores for each of the seven parameters, for a maximum score of 17.

### ELISA

Colon segments (100–200 mg of tissue) were washed with cold phosphate-buffered saline (PBS) and shaken at room temperature in Roswell Park Memorial Institute medium (RPMI) containing 50 µg/mL gentamycin for 30 min at 280 rpm. The colonic tissue fragments were distributed (0.05 g per well) into 24-well flat-bottomed culture plates and incubated in RPMI 1640 medium with 5% fetal bovine serum and 50 µg/mL gentamycin for 24 h at 37 °C. After incubation, the supernatants were collected, spun by centrifugation at 842 g at 4 °C for 15 min, and stored at −70 °C until analysis. IL-12p40, IFN-γ, and IL-6 were measured in colonic culture supernatants by a commercially available ELISA (OptEIA kit, BD Biosciences, USA).

### Isolation of ILC subpopulations and flow cytometry

Cell suspensions were prepared from the colonic lamina propria and spleen as previously described^19,33^. To isolate mononuclear cells from the intestinal lamina propria, gut fragments were cut open and washed three times with PBS by vigorous shaking. Washed gut pieces were subsequently cut into pieces 1–2 cm in length and incubated for 30 min at 37 °C in PBS containing 5% fetal bovine serum, 500 mM EDTA, 1 M HEPES, 100 mM sodium pyruvate, and 1X penicillin/streptomycin. Tissue pieces were washed three times in warm PBS by vigorous shaking and then incubated for 30 min with fresh medium containing collagenase D (1 mg/mL; Roche) and DNase I (1 mg/mL; Roche). The remaining intestinal fragments were filtered through the 100-μm mesh, and the cell suspensions were spun by centrifugation at 431 g for 8 min. After discarding the supernatant, the cell pellets were resuspended in 75% (wt/vol) Percoll (GE Healthcare). Next, 40% (wt/vol) Percoll was added to the suspension. After spinning by centrifugation for 20 min at 1,350 *g*, mononuclear cells were collected from the 75%/40% interphase. To obtain single spleen cells, the spleens were cut into pieces 5 mm in length and incubated for 30 min at 37 °C in RPMI containing 5% FBS and 1% penicillin/streptomycin. The pieces were then pressed through 100-μm mesh. All cells were first preincubated with monoclonal antibody 2.4G2 (anti-mouse CD16/CD32 mAb; BD Pharmingen) to block Fcγ receptors, after which the cells were washed and incubated for 40 min with the appropriate monoclonal antibody conjugates. Incubations were performed in a total volume of 100 μL PBS containing 2 mM EDTA and 2% (vol/vol) bovine serum. Cells were analyzed on an FACSCanto II instrument (BD Biosciences) with FlowJo software (TreeStar). Cells were sorted with a FACSAria III Cell Sorting System to a purity of 95–98%.

The following antibodies were used for flow cytometry: anti-mouse lineage antibodies conjugated to FITC, anti-RORγt antibodies conjugated to APC, anti-NKp46 antibodies conjugated to PE, and anti-CD4 antibodies conjugated to PE (eBioscience).

### Quantitative real-time polymerase chain reaction

Colon tissues were obtained from *Rag2*^−/−^ mice at 9 weeks after the CD4^+^CD45RB^hi^ T cell transfer. Total RNA was extracted using QIAzol Lysis Reagent (QIAGEN, Valencia, CA), dissolved in diethylpyrocarbonate (DEPC)-treated water, and quantified using a Biospec-nano spectrophotometer (Life Science, Columbia, MD). A TOPscript™ cDNA synthesis kit (Enzynomics, Daejeon, Republic of Korea) was used for cDNA synthesis. Quantitative real-time PCR was performed with a 7500 Real-Time PCR System (Applied Biosystems, Foster City, CA) using TOPreal™ qPCR 2X PreMIX (Enzynomics) and a final volume of 25 μL reactions. All reactions were performed in duplicate in a 96-well plate using the following cycling conditions: 40 cycles of 95 °C for 30 s, 63 °C for 30 s, and 68 °C for 1 min. The relative expression of each transcript was calculated with the 2^−*Δ ΔCT*^ method using β-actin levels for normalization. The sequences of the primers used for PCR were as follows: *Il12p40* forward: CGCAAGAAAGAAAAGATGAAGGAG, *Il12p40* reverse: TTGCATTGGACTTCGGTAGATG; *Ifng* forward: CTTCCTCATGGCTGTTTCTGG, *Ifng* reverse: ACGCTTATGTTGTTGCTGATGG; *Il6* forward: ATGGATGCTACCAAACTGGAT, *Il6* reverse: TGAAGGACTCTGGCTTTGTCT; *Il23p19* forward: GTCACTAAGAACTAACAGGACTACCA, *Il23p19* reverse: TGAAAAGTTCCCTTCCCACTT; *Il17a* forward: GGTCAACCTCAAAGTCTTTAACTC, *Il17a* reverse: TTAAAAATGCAAGTAAGTTTGCTG; and *Il22* forward: TTGAGGTGTCCAACTTCCAGCA, *Il22* reverse: AGCCGGACGTCTGTGTTGTTA. The housekeeping control gene *β-actin* was used as an internal control.

### Statistical analyses

Continuous and ordinal variables, including body weight, intestinal inflammation score, mRNA expression level, and protein expression level, are expressed as means with standard errors. A two-tailed Student’s *t*-test was used to compare means between groups. For comparisons of longitudinal data such as body weight changes, repeated measures ANOVA was performed. A *P*-value of < 0.05 was considered to denote a significant difference. All statistical analyses were conducted using R statistical software (version 3.6.0; R Foundation for Statistical Computing, Vienna, Austria).

## Supplementary information

Supplementary information.
