# Supplementary material for: Role of innate lymphoid cells in chronic colitis during anti-IL-17A therapy
Source: Sci Rep. 2020 Jan 15;10:297. doi: 10.1038/s41598-019-57233-w (PMC6962146; doi:10.1038/s41598-019-57233-w)
Supplement: Supplementary file 1 — Supplementary information. [file 41598_2019_57233_MOESM1_ESM.pdf]

Role of innate lymphoid cells in chronic colitis during anti-IL-17A therapy

Chan Hyuk Park[1], A-reum Lee[2], Sang Bong Ahn [3], Chang Soo Eun[4], Dong Soo Han[5]

[1]Department of Internal Medicine, Hanyang University Guri Hospital, Hanyang University College of Medicine, Guri, Korea

[2]Department of Internal Medicine, Hanyang University Guri Hospital, Hanyang University College of Medicine, Guri, Korea

[3]Department of Internal Medicine, Nowon Eulji Medical Center, Eulji University, Seoul, Korea

[4]Department of Internal Medicine, Hanyang University Guri Hospital, Hanyang University College of Medicine, Guri, Korea

[5]Department of Internal Medicine, Hanyang University Guri Hospital, Hanyang University College of Medicine, Guri, Korea

Corresponding author: Dong Soo Han

Department of Internal Medicine, Hanyang University Guri Hospital, Hanyang University College of Medicine

153 Gyeongchun-ro, Guri, Republic of Korea

Postal code: 11923

Phone: +82-31-560-2226

Fax: +82-31-553-7369

E-mail: hands@hanyang.ac.kr

Supplementary information

Figure S1. Expression of inflammatory cytokines including IL-12 (A), IFN-γ (B), and IL-6 (C) in T cell transfer-induced colitis (*Rag2*$^{-/-}$ mice without T cell transfer: n=13; *Rag2*$^{-/-}$ mice with T cell transfer from wild-type mice: n=8; *Rag2*$^{-/-}$ mice with T cell transfer from *Il17a*$^{-/-}$ mice: n=8).

* $P < 0.05$.

NS, not significant.

Figure S2. Flow cytometry analysis of spleen cells in *Rag2*$^{-/-}$ mice (A), *Rag2*$^{-/-}$ mice that received T cells from wild-type mice (B), and *Rag2*$^{-/-}$ mice that received T cells from *Il17a*$^{-/-}$ mice (C).

FSC, forward scatter; SSC, side scatter.

Figure S3. Flow cytometry analysis of colonic lamina propria lymphocytes in the DSS-induced colitis models between wild-type mice and *Il17a*$^{-/-}$ mice. (A) No acute colitis in wild-type mice, (B) no acute colitis in *Il17a*$^{-/-}$ mice, (C) DSS-induced colitis in wild-type mice, and (D) DSS-induced colitis in *Il17a*$^{-/-}$ mice.

Flow cytometry analysis was performed on the 8th day after the DSS treatment.

DSS, dextran sulfate sodium

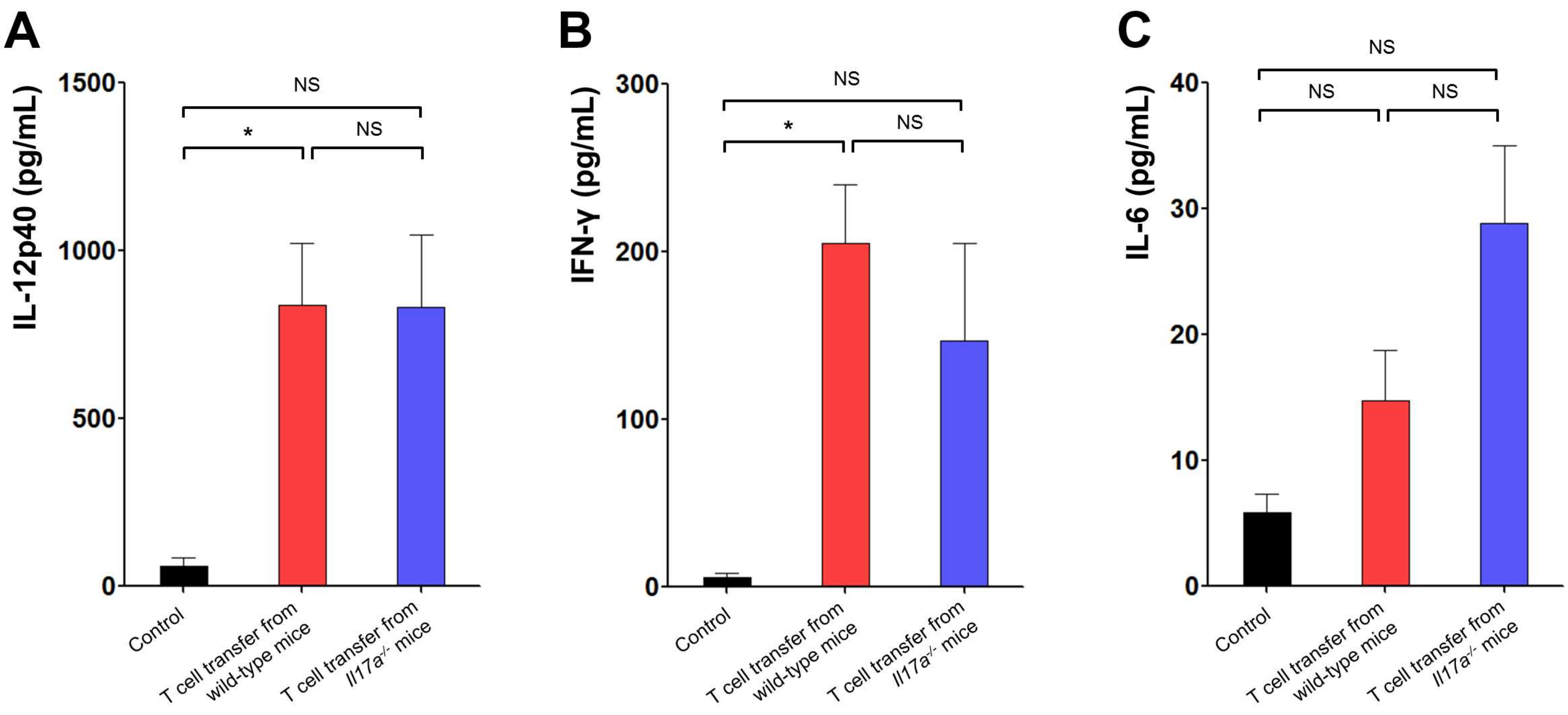

Figure S1. Expression of inflammatory cytokines including IL-12 (A), IFN-γ (B), and IL-6 (C) in T cell transfer-induced colitis (*Rag2*$^{-/-}$ mice without T cell transfer: n=13; *Rag2*$^{-/-}$ mice with T cell transfer from wild-type mice: n=8; *Rag2*$^{-/-}$ mice with T cell transfer from *Il17a*$^{-/-}$ mice: n=8).

* P <0.05.

NS, not significant.

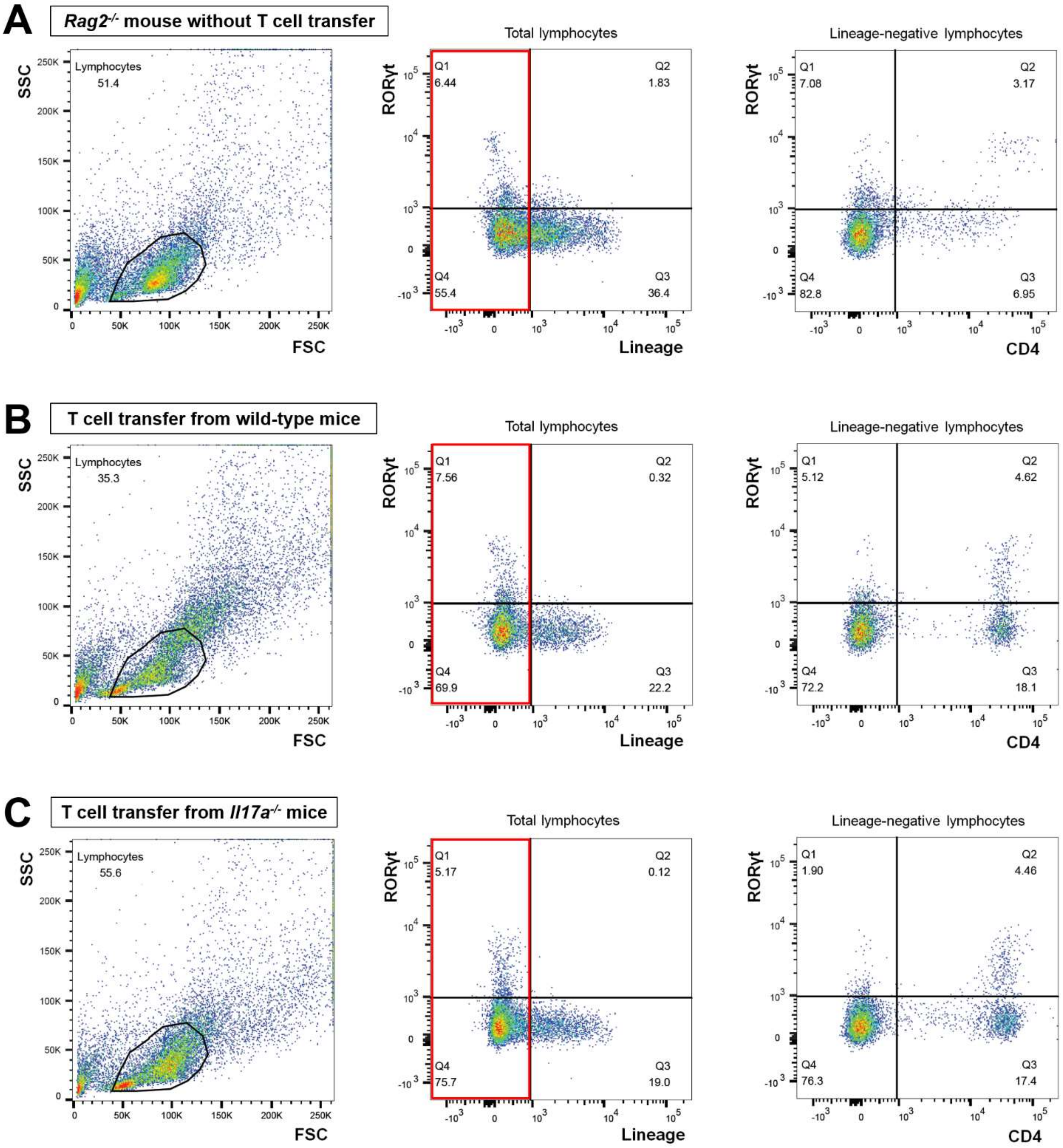

Figure S2. Flow cytometry analysis of spleen cells in *Rag2$^{-/-}$* mice (A), *Rag2$^{-/-}$* mice that received T cells from wild-type mice (B), and *Rag2$^{-/-}$* mice that received T cells from *Il17a$^{-/-}$* mice (C).

FSC, forward scatter; SSC, side scatter.

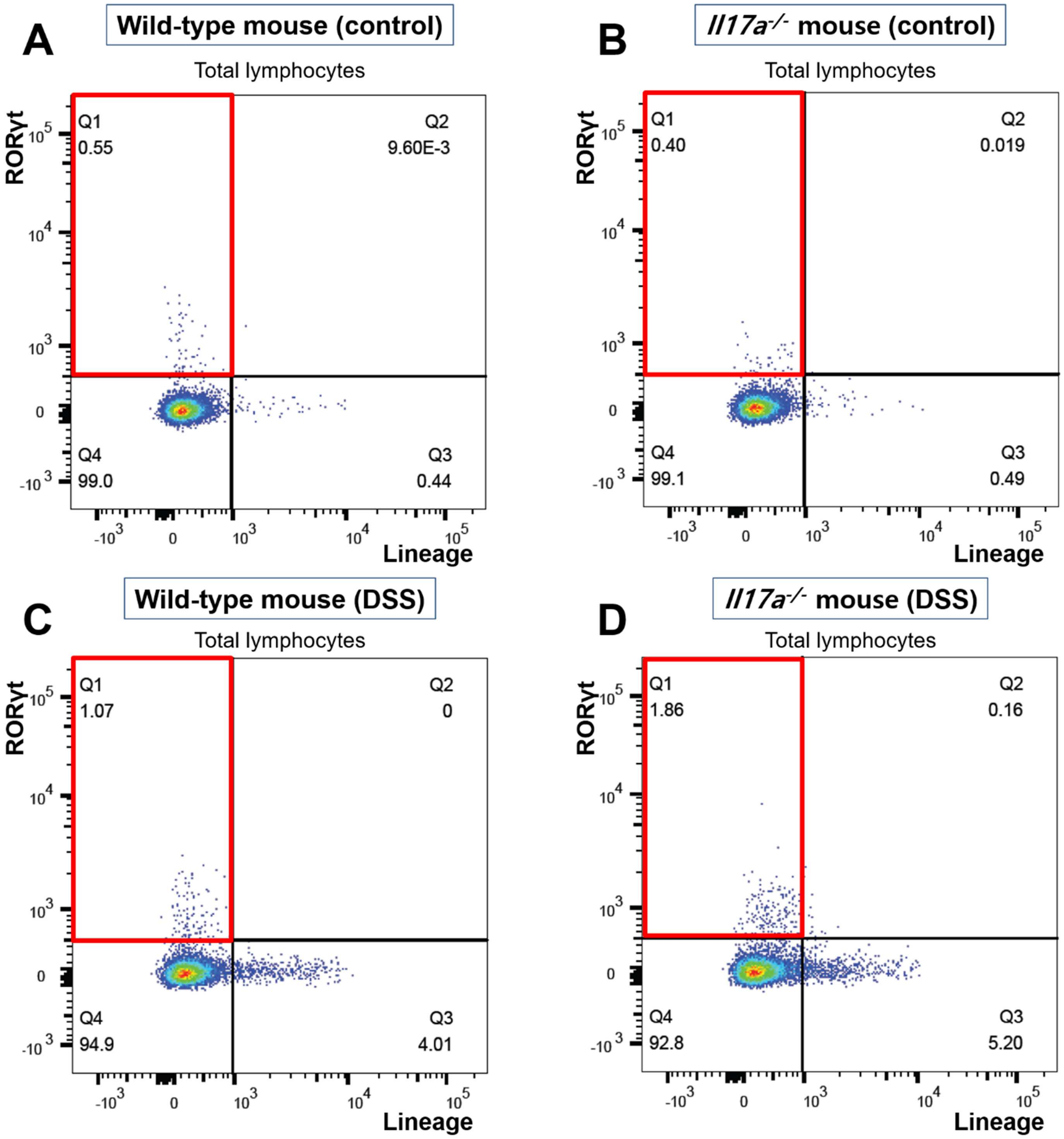

Figure S3. Flow cytometry analysis of colonic lamina propria lymphocytes in the DSS-induced colitis models between wild-type mice and *Il17a*$^{-/-}$ mice. (A) No acute colitis in wild-type mice, (B) no acute colitis in *Il17a*$^{-/-}$ mice, (C) DSS-induced colitis in wild-type mice, and (D) DSS-induced colitis in *Il17a*$^{-/-}$ mice.

Flow cytometry analysis was performed on the 8th day after the DSS treatment.

DSS, dextran sulfate sodium
